# Hurricane-Associated Mold Exposures Among Patients at Risk for Invasive Mold Infections After Hurricane Harvey — Houston, Texas, 2017

**DOI:** 10.15585/mmwr.mm6821a1

**Published:** 2019-05-31

**Authors:** Nancy A. Chow, Mitsuru Toda, Audrey F. Pennington, Enock Anassi, Robert L. Atmar, Jean M. Cox-Ganser, Juliana Da Silva, Bobbiejean Garcia, Dimitrios P. Kontoyiannis, Luis Ostrosky-Zeichner, Lauren M. Leining, Jennifer McCarty, Mayar Al Mohajer, Bhavini Patel Murthy, Ju-Hyeong Park, Joann Schulte, Jennifer A. Shuford, Kimberly A. Skrobarcek, Samantha Solomon, Jonathan Strysko, Tom M. Chiller, Brendan R. Jackson, Ginger L. Chew, Karlyn D. Beer

**Affiliations:** ^1^Division of Foodborne, Waterborne, and Environmental Diseases, National Center for Emerging and Zoonotic Infectious Diseases, CDC; ^2^Epidemic Intelligence Service, CDC; ^3^Division of Environmental Health Science and Practice, National Center for Environmental Health, CDC; ^4^Harris Health System, Houston, Texas; ^5^Baylor College of Medicine, Houston, Texas; ^6^Respiratory Health Division, National Institute for Occupational Safety and Health, CDC; ^7^Division of Global HIV and Tuberculosis, Center for Global Health, CDC; ^8^Texas Department of State Health and Services; ^9^The University of Texas MD Anderson Cancer Center, Houston, Texas; ^10^McGovern Medical School/Memorial Hermann-Texas Medical Center, Houston Texas; ^11^UTHealth School of Public Health, Houston, Texas; ^12^Baylor St. Luke’s Medical Center, Houston, Texas; ^13^Division of State and Local Readiness, Center for Preparedness and Response, CDC; ^14^Houston Health Department, Houston, Texas; ^15^Division of Healthcare Quality Promotion, National Center for Emerging and Zoonotic Infectious Diseases, CDC; ^16^Harris County Public Health, Houston, Texas; ^17^Public Health Associate Program, Center for State, Tribal, Local, and Territorial Support, CDC.

In August 2017, Hurricane Harvey caused unprecedented flooding and devastation to the Houston metropolitan area (*1*). Mold exposure was a serious concern because investigations after Hurricanes Katrina and Rita (2005) had documented extensive mold growth in flood-damaged homes (*2*,*3*). Because mold exposure can cause serious illnesses known as invasive mold infections (*4*,*5*), and immunosuppressed persons are at high risk for these infections (*6*,*7*), several federal agencies recommend that immunosuppressed persons avoid mold-contaminated sites (*8*,*9*). To assess the extent of exposure to mold and flood-damaged areas among persons at high risk for invasive mold infections after Hurricane Harvey, CDC and Texas health officials conducted a survey among 103 immunosuppressed residents in Houston. Approximately half of the participants (50) engaged in cleanup of mold and water-damaged areas; these activities included heavy cleanup (23), such as removing furniture or removing drywall, or light cleanup (27), such as wiping down walls or retrieving personal items. Among immunosuppressed persons who performed heavy cleanup, 43% reported wearing a respirator, as did 8% who performed light cleanup. One participant reported wearing all personal protective equipment (PPE) recommended for otherwise healthy persons (i.e., respirator, boots, goggles, and gloves). Immunosuppressed residents who are at high risk for invasive mold infections were exposed to mold and flood-damaged areas after Hurricane Harvey; recommendations from health care providers to avoid exposure to mold and flood-damaged areas could mitigate the risk to immunosuppressed persons.

Interviews were conducted with a convenience sample of immunosuppressed residents from three hospital systems in the Houston metropolitan area. Eligible residents were selected because of risk factors for invasive mold infections (*7*); participants included persons who had received a solid organ transplant in the past year or who had been prescribed an immunosuppressive medication, including tumor necrosis factor inhibitors, cyclosporine, or chemotherapeutic agents, in the last 3 months. Models developed by CDC’s Geospatial Research, Analysis and Services Program were used to predict whether residents’ homes had been flooded. Residents whose homes were predicted to have been flooded were prioritized for contact.

Among the three hospital systems from which participants were selected, systematic, hospital-wide messaging about avoiding mold exposure had not been disseminated before Hurricane Harvey. A CDC questionnaire developed after Hurricanes Katrina and Rita was modified and field-tested. Questions were focused on experiences with housing, flooding and mold, cleanup activities, and PPE. Cleanup was categorized as either heavy (e.g., removing furniture, drywall, or carpeting) or light (e.g., sweeping, wiping off counters or walls, or retrieving personal items). The 20-minute questionnaire was administered by telephone in either English or Spanish by trained interviewers during October 21–November 8, 2017; no personally identifiable information was collected. Interviewers were trained on types of PPE. Federal agency–developed materials on how residents who are not immunosuppressed can safely conduct mold cleanup after disasters were made available to participants after the interview. Because this work was part of an ongoing public health response, it was determined to be nonresearch public health practice by CDC’s Human Research Protection Office and the local hospitals and thus was not subject to institutional review board review.

Interviewers attempted to contact 800 immunosuppressed persons, and 167 (21%) were reached, 109 (65%) of whom consented to be interviewed. Among these 109 persons, 103 (94%) had stayed within the Houston metropolitan area during Hurricane Harvey (August 25–August 29, 2017) or in the months afterwards (August 30–interview date); the survey sample consisted of these 103 persons. During the hurricane, 77 (75%) reported staying at home for the majority of the time, 20 (19%) stayed at friends or relatives’ homes, and seven (7%) stayed at other places, including hotels, hospitals, shelters, or nursing homes (Table 1). In contrast, since the hurricane, 83 (81%) stayed at home, 12 (12%) stayed at friends or relatives’ homes, and six (6%) stayed at other places. Of the 102 participants whose responses were available, 92 (89%) reported living in their homes at the time of the interview; of the 10 who did not, eight had been back to their homes. One participant’s response was not available.

**TABLE 1:** Housing status of immunosuppressed survey participants (N = 103) who reported staying in the Houston metropolitan area before or since Hurricane Harvey — Houston, Texas, August–November 2018

Housing status	No. (%)
During Hurricane Harvey*	
Home	77 (75)
Friends or relatives	20 (19)
Other^†^	7 (7)
Since Hurricane Harvey*	
Home	83 (81)
Friends or relatives	12 (12)
Other^†^	6 (6)
**Currently living at home**	
Yes	92 (89)
No	10 (10)
Reentered home	8 (80)
Did not reenter home	2 (20)
Response not available	1 (1)

* Participants might have provided more than one response or provided a response not shown in the options given; thus, the subtotals do not sum to 103.

**^†^** Hospital, hotel, nursing home, or shelter.

Forty-six (45%) respondents reported that water had entered their homes, 37 (80%) of whom reported that the first floor living space was flooded with a median of 3 inches of water (interquartile range [IQR] = 1–12 inches) for a median of 3 days (IQR = 1–4 days) (Table 2). Among the 37 respondents who reported water in their living space, 28 (76%) reported seeing or smelling mold inside the home after the hurricane, and 32 (86%) had their home cleaned for water damage or remediated for mold. Seventeen (53%) participants lived in the house during cleanup, and 17 (53%) performed the cleanup themselves. In addition, 23 (62%) reported plans for cleanup or remediation within the next 6 months.

**TABLE 2:** Flooding, mold, and cleanup experiences of immunosuppressed survey respondents who reported water entry into the first floor living space after Hurricane Harvey (N = 37) — Houston, Texas, August–November 2018

Experiences and plans	No. (%)
**Saw or smelled mold**	
Yes	28 (76)
No	8 (22)
Don’t know	1 (3)
**Cleaned or remediated home**	
Yes	32 (86)
Lived in home during cleanup	17 (53)
Did not live in home during cleanup	15 (47)
Who completed the cleanup*	
Self	17 (53)
Friends or family	12 (38)
Professional remediation	15 (47)
No	5 (14)
**Plans for cleaning or remediation within 6 mos**	
Yes	23 (62)
No	11 (30)
Don’t know	3 (8)

* Participants might have provided more than one response; thus, the subtotals do not sum to 32.

Participation in cleanup activities for any home was assessed among all 103 survey respondents; overall, 50 (49%) engaged in any cleanup activities, including 23 (22%) who engaged in heavy cleanup activities for a median of 7 days (IQR = 5–14 days) and 27 (26%) who participated in only light cleanup activities for a median of 4 days (IQR = 2–14 days) (Table 3). Among the 23 participants who engaged in heavy cleanup activities, 10 (43%) wore a full-face, half-face, or N-95 respirator,* half of whom reported always wearing a respirator during cleanup. Eighteen participants wore gloves during heavy cleanup, eight wore boots, and two wore goggles. Three participants reported using no PPE. Among the 27 participants who engaged in light cleanup activities, two wore a respirator, both of whom reported wearing it at all times during cleanup. Thirteen wore gloves, two used goggles, and one wore boots; seven used no PPE. Among all participants who engaged in cleanup activities, only one wore all PPE recommended for otherwise healthy persons.

**TABLE 3:** Time spent in cleanup activities and use of personal protective equipment (PPE) during cleanup among immunosuppressed respondents who stayed in Houston during and after Hurricane Harvey (N = 103) — Houston, Texas, August–November 2018

Time spent cleaning up and PPE use	Heavy cleanup*	Light cleanup^†^	Total
	(n = 23)	(n = 27)	(n = 50)
	No. (%)	No. (%)	No. (%)
**Hrs per day engaged in cleanup**			
<1	0 (0)	13 (48)	**13 (26)**
1–4	8 (35)	9 (33)	**17 (34)**
5–7	9 (39)	0 (0)	**9 (18)**
≥8	6 (26)	5 (19)	**11 (22)**
**Wore a mask^§^**			
Always	5 (22)	2 (7)	**7 (14)**
Most of the time	1 (4)	0 (0)	**1 (2)**
Less than half of the time	4 (17)	0 (0)	**4 (8)**
No	13 (57)	23 (85)	**36 (72)**
Missing information	0 (0)	2 (7)	**2 (4)**
**Wore boots**			
Yes	8 (35)	1 (4)	**9 (18)**
No	15 (65)	26 (96)	**41 (82)**
**Wore gloves**			
Yes	18 (78)	13 (48)	**31 (62)**
No	5 (22)	14 (52)	**19 (38)**
**Wore goggles**			
Yes	2 (9)	2 (7)	**4 (8)**
No	21 (91)	25 (93)	**46 (92)**
**Wore any PPE**			
Yes	20 (87)	20 (74)	**40 (80)**
No	3 (13)	7 (26)	**10 (20)**

* For example, removing furniture, drywall, or carpeting.

**^†^** For example, sweeping, wiping off counters or walls, or retrieving personal items.

^§^ Mask includes full-face respirator, half-face respirator, or N-95 respirator.

Among all 103 participants, 62 (60%) reported hearing or reading about what to wear to clean up mold and floodwater. The most commonly reported information sources included television (14), word of mouth (14), and health care providers (seven). No participants reported obtaining information from social media or a website.

## Discussion

This investigation of mold exposures and PPE use after Hurricane Harvey found that a convenience sample of immunosuppressed adult residents were exposed to mold and water-damaged areas. Immunosuppressed persons are at risk for invasive mold infections (primarily respiratory) with mortality rates as high as 50% (*6*). Although federal agencies recommend that immunosuppressed persons avoid flooded and mold-contaminated buildings (*9*), approximately half of survey participants engaged in cleanup activities, with approximately half of those who engaged in heavy cleanup and most of those engaged in light cleanup reporting not wearing respiratory protection; gloves were the most frequently reported PPE used.

In disaster settings such as Hurricane Harvey, immunosuppressed residents might experience difficulty in adhering to recommendations about avoiding mold-contaminated sites if the majority of homes in the community are affected. In such cases, proper use of an appropriate respirator and other PPE during reentry to the home might reduce mold exposure.

No participants reported websites or social media as sources of information about what one should wear during cleanup of mold and floodwater. This could reflect a unique demographic profile in this group; however, the profile could not be assessed because demographic information was not obtained as part of the survey.

The findings in this report are subject to at least three limitations. First, survey participants were not representative of all immunosuppressed patients in the Houston area because the participants consisted of a convenience sample of patients with specific conditions from three hospital systems. Although these findings cannot be extrapolated to all immunosuppressed residents in the Houston area during and after Hurricane Harvey, they suggest that a substantial number of immunosuppressed persons were exposed to mold and flood-damaged areas and that PPE use among some immunosuppressed persons who engaged in cleanup activities was low. Second, although it was ascertained that the three hospital systems had not conducted systematic, hospital-wide messaging about avoiding mold exposure before Hurricane Harvey, survey participants were not asked whether they had been told by a health care provider to avoid exposure to mold. Thus, it was not possible to determine whether survey participants who were exposed to mold were aware of federal recommendations. Finally, eligibility criteria included immunosuppressive medications for health conditions of varying severity, and information on specific conditions was not collected. For example, some participants were prescribed a tumor necrosis factor inhibitor for rheumatoid arthritis, whereas others received cyclosporine for a solid organ transplant. It is possible that immunosuppressed persons in better physical health were more likely to consent to the survey. As a result, these findings might overestimate the percentage of immunosuppressed persons performing cleanup activities.

Among a sample of immunosuppressed Houston area residents, many were exposed to mold and flood-damaged homes after Hurricane Harvey. Many residents at high risk for invasive mold infections engaged in activities to clean up mold and flood-damaged areas without wearing PPE recommended for otherwise healthy persons. Although recommendations for immunosuppressed persons are to avoid mold-contaminated sites, these findings might help prompt future studies on the knowledge, attitudes, and practices of PPE use among immunosuppressed persons in posthurricane settings and other locations experiencing flooding when complete avoidance of mold-contaminated sites is difficult. In turn, these studies could help inform future decisions about PPE recommendations for this population.

**Summary**

**What is already known about this topic?**

Immunosuppressed persons are at risk for invasive mold infections and should avoid exposures such as those present during hurricane and flood cleanup activities.

**What is added by this report?**

Among a convenience sample of immunosuppressed residents in the Hurricane Harvey-affected area of Houston, Texas, 49% engaged in cleanup activities in water-damaged or mold-contaminated homes. Use of respiratory protection was low.

**What are the implications for public health practice?**

Health care providers should advise immunosuppressed persons to avoid exposure to water-damaged and mold-contaminated areas to reduce their risk for invasive mold infections.

## Footnotes

* The N95 respirator is the most common particulate-filtering facepiece respirator and filters at least 95% of airborne particles. https://www.cdc.gov/niosh/npptl/topics/respirators/disp_part/n95list1.html.
